# Enhanced replication of avian-origin H3N2 canine influenza virus in eggs, cell cultures and mice by a two-amino acid insertion in neuraminidase stalk

**DOI:** 10.1186/s13567-016-0337-x

**Published:** 2016-05-09

**Authors:** Yan Lin, Xing Xie, Yanbing Zhao, Dildar Hussain Kalhoro, Chengping Lu, Yongjie Liu

**Affiliations:** College of Veterinary Medicine, Nanjing Agricultural University, Nanjing, 210095 China; College of Animal Science and Technology, Nanjing Agricultural University, Nanjing, 210095 China

## Abstract

Canine influenza virus (CIV) is a newly identified, highly contagious respiratory pathogen in dogs. Recent studies indicate that avian-origin H3N2 CIV are circulating in Chinese dogs. To investigate the effects of a two-amino acid (2-aa) insertion naturally occurring at the distal end of the neuraminidase (NA) stalk found in Chinese isolates since 2010 on virus replication and virulence, we rescued the CIV strain, A/canine/Jiangsu/06/2011(H3N2) and its NA mutant without the 2-aa insertion using reverse genetics. The NA stalk length affected virus growth in cell culture. Compared to the short stalk strain (without 2-aa insertion), the long stalk strain (with 2-aa insertion) exhibited higher peak titers and greater yields in Madin-Darby canine kidney (MDCK) cells, chicken embryo fibroblasts and canine bronchiolar epithelial cells, as well as much larger plaques in MDCK cell monolayers. Furthermore, mice inoculated with the long stalk strain showed more severe pathologic damage in lung and higher proportion of detectable viral RNA in tissues. The long stalk strain induced local IFN-γ production with faster kinetics and higher levels in mice. However, in chickens, the two viral strains showed no significant difference with nearly the same proportion of detectable viral RNA loads in tissues. These observations suggest that the 2-aa insertion in the NA stalk acquired by avian-origin H3N2 CIV helps to enhance viral replication and is likely a result of adaptive evolution in canine hosts.

## Introduction

Influenza A viruses (IAV) are important pathogens in both mammalian and avian hosts, and interspecies transmission of this virus is a crucial feature of its ecology and epidemiology [1]. In 2004, an equine-origin H3N8 influenza virus was first isolated from racing greyhounds with serious respiratory disease in Florida [2]. Frequent outbreaks were subsequently reported, and the infection rapidly disseminated across the United States. In 2007, a different influenza virus, subtype H3N2, caused an outbreak of canine respiratory disease in South Korea [3]. The H3N2 CIV appeared to be entirely of avian origin, but it was able to be transmitted between dogs [4]. The serological surveillance in South Korea and southern China showed that the seroconversion rates for H3N2 IAV were approximately 3.3 and 10%, respectively, in the sampled dog population [5, 6]. These surveillance results suggest that H3N2 avian-origin CIV has become endemic in the canine populations in South Korea and China.

IAV interact with their hosts mostly through two critical glycoproteins, hemagglutinin (HA) and neuraminidase (NA). HA recognizes receptors on target cells, and NA cleaves sialic acids from receptors, preventing self-aggregation and facilitating the release of virus during budding from host cells [7, 8]. The NA stalk region varies considerably in length, even within the same subtypes, and plays a role in replication and pathogenesis [9]. Coordination of the NA stalk with HA is known to play a significant role in virus growth and adaption to the host [10, 11].

In the first few years after the outbreak in 2007, CIV isolated from South Korea and Guangdong, China, only possessed 40 amino acids (aa) in the NA stalk. However, in 2010, a 2-aa insertion was found at the distal end of the NA stalk in all six isolates from Jiangsu province, China [12]. Since then, all isolates from different provinces of China, such as Zhejiang [13], Beijing and Liaoning [14], have been shown to possess the insertion. In 2012, the insertion was also found in a Thailand H3N2 CIV isolate [15]. This genetic modification in the NA protein might be an evolutionary adaptation of avian influenza virus (AIV) to dogs. Deletions in the stalk region of NA have been found frequently in AIV in poultry [16, 17], but few studies have addressed the insertion mutations in the NA stalk as a result of virus evolution. Therefore, the aim of this study was to determine the biological properties of this 2-aa insertion generated in nature. This evolutionary change may give rise to a better understanding of the mutational frequencies associated with influenza virus replication in the canine host.

## Materials and methods

### Virus strains, cells and medium

A/canine/Jiangsu/06/2011(H3N2) identified from pet dogs in the Jiangsu province of China [12] was used as the wild-type canine influenza virus in this study. Primary cultures of chicken embryo fibroblasts (CEF) were prepared from specific-pathogen-free (SPF) chicken embryos (10 days old) as described previously [18]. Primary canine bronchiolar epithelial cells (CBE) was prepared from beagles according to protocols previously described [19]. Madin-Darby canine kidney (MDCK) cells, CEF cells and 293T cells were cultured in Dulbecco’s modified essential medium (DMEM) whereas CBE cells were cultured in DF12, and all cells were maintained at 37 °C and 5% (v/v) CO_2_ atmosphere.

### Animals

The 10-day-old SPF chicken embryos and SPF White Leghorn chickens (40 days old) were purchased from the Experimental Animal Center, Jiangsu Academy of Agricultural Sciences. BALB/c mice (7 weeks old, female) were purchased from the Animal Experiment Center, Yangzhou University. All animal experiments complied with the guidelines of the Animal Welfare Council of China, and approval was obtained from the Animal Ethics Committee of Nanjing Agricultural University.

### Sequence analysis

The NA gene sequences of 10 reference strains of CIV from China (south, east, and northeast), South Korea and Thailand between 2007 and 2013 were compared in this study. Reference sequences were obtained from the National Center for Biotechnology Information (NCBI). Comparisons of aa sequences were made using DNASTAR software.

### Site-directed mutagenesis and virus generation

A site-directed mutagenesis kit (TaKaRa, Dalian, China) was used to create specific mutation in the NA gene. H3N2 CIV with a long-stalk NA (wild-type virus) or a short-stalk NA (delNA virus) were rescued by an eight-plasmid reverse genetics system as previously described [20] and designated as r-06 and r-06/NA2 virus, respectively.

### Replication of viruses in eggs

Ten-day-old embryonated chicken eggs were inoculated with 200 μL of virus stock serially diluted from 10^−1^ to 10^−9^ in phosphate-buffered saline (PBS). Eggs were incubated at 37 °C for 72 h before allantoic fluid was harvested and tested by hemagglutination (HA) assays at room temperature using 0.75% chicken RBC. Fifty-percent egg infective dose (EID_50_) titers were calculated by the Reed–Muench method with eight eggs per dilution. To further measure virus quantity, plaque assays were performed on monolayers of MDCK cells in 12-well tissue culture plates. Serial dilutions were prepared from allantoic fluid collected above, and 500 μL of each dilution was incubated on monolayers in duplicate for 1 h at 37 °C and 5% CO_2_. Thereafter, the monolayers were overlaid with a mixture of 2% agarose and cell culture medium containing 0.8 μg/mL TPCK-treated trypsin. Cultures were incubated for 3 days at 37 °C, fixed with 4% formaldehyde and stained with 1% crystal violet to reveal plaques. Experiments were performed three times with different preparation of viruses.

### NA activity assays

For enzymatic assays, 50 μL of a small soluble substrate 4-methylumbelliferyl N-acetylneuraminic acid (4-MU-NANA; Sigma, Beijing, China) was serially diluted in triplicate at concentrations from 5 to 120 μM in black U-bottomed 96-well microtiter plates, and then 50 μL virus with an equivalent titer of 2 × 10^5^ pfu/mL was added to each well. The plates were incubated at 37 °C, and UV fluorescence emission was measured every 5 min, for 60 min, with a 355-nm excitation filter and 460-nm emission filter. The Michaelis–Menten constant (*K*_m_) and maximum velocity of reaction (*V*_max_) were calculated by the Michaelis–Menten equation. Three different preparations of the two viruses were tested in three different experimental runs to determine the mean values.

### Virus replication kinetics in cell culture

Confluent CEF and CBE cell monolayers in 12-well tissue culture plates were inoculated with virus at the multiplicity of infection (MOI) of 0.01 pfu/cell, while MDCK cells were infected with an MOI of 0.001 pfu/cell. The MOI were selected on the basis of a preliminary sighting study, in which the starting MOI was screened from the fixed levels of 0.001, 0.01 and 0.1 pfu/cell as an MOI expected to examine the viral growth during 72 h infection process. Then all three cells were cultured with 1 mL DMEM with l-1-tosylamide-2-phenylethyl chloromethyl ketone (TPCK)-treated trypsin (0.8 μg/mL for MDCK, and 0.15 μg/mL for CEF and CBE, as indicated by preliminary data). Supernatants were collected 12, 24, 36, 48, 60 and 72 h post-infection and titrated by plaque assay in MDCK cells. Experiments were performed three times with different preparation of viruses.

### Immunofluorescence staining and flow cytometry analysis

For immunofluorescence analysis, virus- or mock-infected cells in 12-well tissue culture plates were fixed with 4% paraformaldehyde in PBS at room temperature for 20 min, after which cells were washed with PBS and permeabilized in 0.2% Triton X-100 at room temperature for 5 min. Cells were then incubated at 37 °C for 1 h with rabbit anti-NS1 protein polyclonal serum (prepared in our laboratory using purified NS1 protein) diluted 1/1000 in PBS. Cells were washed with PBS and incubated with secondary FITC-labeled goat anti-rabbit antibodies (Dingguo, Beijing, China) diluted 1/500 in PBS at 37 °C for 1 h. After three washes with PBS, the cells were observed with a fluorescence microscope.

For flow cytometry, virus- or mock-infected cells were digested with 0.25% trypsin–EDTA solution from the tissue culture flask, followed by incubation at 37 °C for 1 h with rabbit anti-NS1 protein polyclonal serum. The subsequent steps were carried out in the same manner as for the immunofluorescence analysis. Finally, flow cytometric analysis was performed using an Accuri C6 flow cytometer (BD Accuri) and analyzed using CFlow plus software (Accuri). Three different preparations of the two viruses were tested in three different experimental runs to determine the mean values.

### Experimental infections in mice and chickens

In the mouse experiment, intranasal inoculations were given to group I (*n* = 30) with r-06 strain, group II (*n* = 30) with r-06/NA2 strain and group III (*n* = 30) with PBS. A total volume of 50 μL of the virus stock was inoculated per mouse with a viral titer of 2 × 10^6^ pfu/mL. The percent of body weight change was calculated from body weights of 10 mice recorded daily after infection relative to that at day 0 (pre-infection). Five mice from each group were euthanized humanely according to a pre-designated schedule. At each of the time points of 2, 4, 6, 8 and 14 days post-infection (dpi), tissues including lung, brain, heart, spleen, liver, intestine and kidney as well as feces were collected.

In the chicken experiment, 3 groups of 20 SPF White Leghorn chickens were intranasally inoculated with a viral titer of 2 × 10^6^ pfu/mL r-06, r-06/NA2 viruses or PBS in a volume of 100 μL. The percent of body weight change was calculated from body weights recorded daily after infection relative to that at day 0 (pre-infection). Five chickens were chosen randomly on 2, 5, 9 and 14 dpi from each group and euthanized humanely. The oropharyngeal and cloacal swabs, heart, liver, spleen, lung, kidney, brain, intestine and pectoralis were collected for virus detection.

### Histopathologic evaluation of lungs

Histopathology was performed as described previously [12]. Lung sections were blindly examined and given an estimated score of the severity of the interstitial pneumonia: 0 = no microscopic lesions; 1 = mild interstitial pneumonia and <10% of the lung affected; 2 = moderate multifocal interstitial pneumonia and 10–40% of the lung affected; 3 = severe interstitial pneumonia and >40% of the lung affected. In section of each mouse, 2 fields were randomly selected for evaluation. Lungs of all five mice from 6 dpi and five chickens from 5 dpi of two infected groups were examined.

### Immunohistochemistry and quantitative assessment of immunostaining

Immunohistochemistry on sections from the lung, spleen and brain was performed as described previously [12]. Tissue sections were blindly examined and given an estimated score of the immunostaining. In the lung and spleen, the extent of the staining was semi-quantitatively scored on a scale of 0 to 3. Score criteria of different scales were assigned based on the percentage of positive staining per examined area: 0, <10%; 1, 10–30%; 2, 30–60%; 3, >60%. The intensities of the signals were graded as 1+ (weak), 2+ (intermediate), and 3+ (strong). Then, a combined score (0–9) for each section was calculated by multiplying the values of these two categories [21]. Cases were classified as negative, 0 points, positive, 1–9 points. In the brain, at least 600 cells (from 3 independent experiments, 200 cells/each) manually counted and the percentage of the positively stained cells was calculated. In each tissue specimen from each mouse, three fields were randomly selected for evaluation. All five mice from 6 dpi of two infected groups were examined.

### Quantitation of viral loads and cytokine levels

Tissues and feces were homogenized in PBS at a ratio of 1:5 (g/mL) and centrifuged at 10 000 × *g* for 30 min. The supernatants were collected for the extraction of viral RNA with the Virus Nucleic Acid Extraction Kit II (Geneaid, Taiwan). Real-time PCR for quantitation of viral loads was performed as described previously [12]. The set of primers was designed based on the region of the matrix gene as follows: TCTATCGTCCCATCAGGC/GGTCTTGTCTTTAGCCATTC. The data were normalised per gram. In addition, supernatants of lung tissue from mice and chickens were also used for the analysis of IFN-γ and TNF-α using the commercially available ELISA kits (Jiancheng, Nanjing, China).

### Statistical analysis

All statistical analyses were performed with Prism 5 software (GraphPad). Data are expressed as arithmetic means ± standard deviations. Comparisons between the two experimental groups were made by a Student’s *t* test with two-tailed analysis, but cytokine data among control and two experimental groups were analyzed using one-way ANOVA with a Bonferroni multiple-comparison test. *P* values of less than 0.05 were considered statistically significant [22].

## Results

### Sequence alignment of NA stalk region of H3N2 CIV from different geographical areas

**Figure 1:**
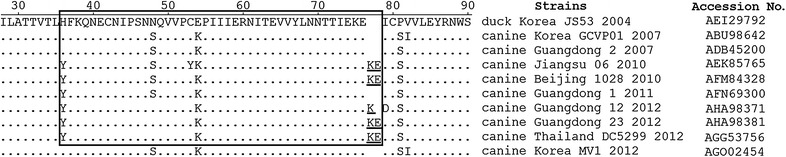
**Alignment of NA stalk region of H3N2 CIV isolates from different areas from 2007 to 2012.** The Clustal W method was used for the alignment, and boxed residues represent the NA stalk regions (aa 36–78). The consensus is the most similar avian isolate. Identical residues are indicated by dots, and underline residues denote the insertion in the stalk.

The NA stalk sequences of reference strains were obtained from NCBI, and their accession numbers are listed in Figure 1. The alignment of the stalk region shows that H3N2 CIV from South Korea and Guangdong, China, in 2007 contained only 40 aa in the NA stalk. However, a 2-aa insertion at the distal end of the NA stalk was found widely in viruses isolated from eastern and northeastern China since 2010. In 2012, strains were reported in Guangdong with 1- or 2-aa insertions in NA. Curiously, until 2012, no insertion in the NA stalk was reported in South Korean H3N2 CIV, whereas Thailand had reported a case of an H3N2 CIV containing the insertion (Figure 1).

### Generation of viruses by reverse genetics and their growth in MDCK cells and eggs

To determine whether the two amino acids naturally occurring as inserts in 06 influence viral yield, we rescued the Jiangsu 06 virus (r-06) and its NA mutant with the 2-aa deletion at the distal end of the NA stalk (r-06/NA2). Reassortant viruses were verified by RT-PCR, followed by sequencing of the NA gene and seven other gene segments to confirm there was no other mutation except the 22-aa deletion in NA genes of r-06/NA2 virus.

**Figure 2:**
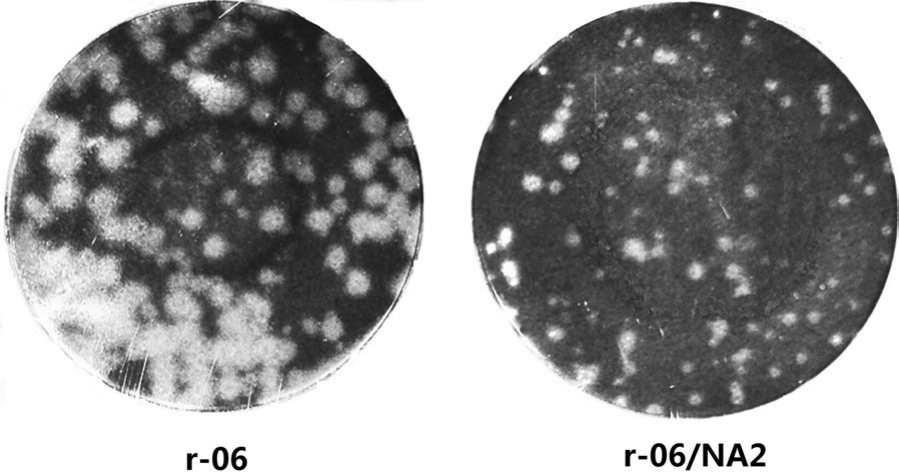
**Plaque morphology of r-06 and r-06/NA2 strains grown in MDCK cell monolayers.** Cells were infected at an MOI of 0.001 and cell monolayers were fixed with formaldehyde at 72 h post-infection and stained with 1% crystal violet.

Growth characteristics of rescued viruses were analyzed in MDCK cells. It was apparent that r-06/NA2 without the 2-aa insertion formed small pin-point plaques on MDCK cell monolayers at 72 h after inoculation, while the r-06 strain made much larger plaques than did the r-06/NA strain (Figure 2). The r-06/NA2 strain formed plaques after 48 h, whereas the r-06 strain formed plaques after only 36 h.

Growth characteristics of rescued viruses were also compared in eggs using an EID_50_ experiment performed with the same viral titer of 2 × 10^6^ pfu/mL. The EID_50_ titer of r-06 was not statistically different from that of r-06/NA2 (10^−7.020 ± 0.098^/200 μL versus 10^−6.793 ± 0.104^/200 μL, *P* > 0.05). However, notably, the HA titer of r-06 was significantly higher than that of r-06/NA2 (2^−7.260 ± 0.061^ versus 2^−5.950 ± 0.038^, *P* < 0.0001) by analysis of allantoic fluid from all infected eggs with 10^−3^–10^−5^ dilutions of the two viruses. Further, we measured plaque forming unit (PFU) counts of the two viruses in the above allantoic fluid using a plaque assay. Similar to an HA titer, the PFU value of r-06 was also 2–4 times higher than that of r-06/NA2 (10^6.318 ± 0.087^ pfu/mL versus 10^5.835 ± 0.120^ pfu/mL, *P* = 0.031). The findings suggest that the 2-aa insertion in the NA stalk did not significantly change virus virulence in eggs, but increased virus yield in eggs.

### Replication kinetics of r-06 and r-06/NA2 viruses in cell cultures

**Figure 3:**
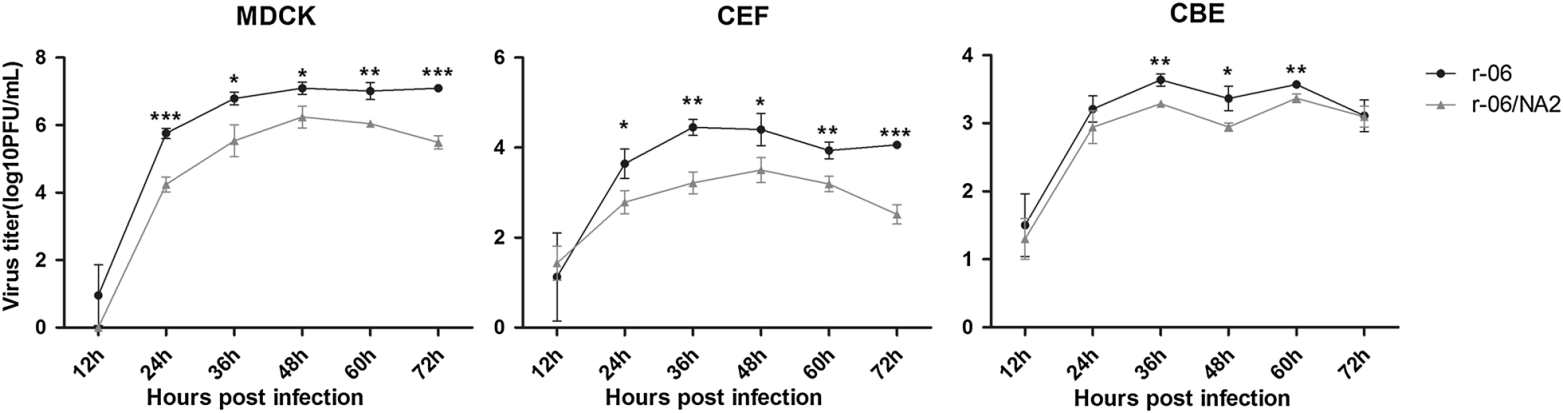
**Replication kinetics of r-06 and r-06/NA2 strains in MDCK, CEF and CBE cell cultures.** MDCK, CEF and CBE cells were infected at an MOI of 0.001, 0.01 and 0.01, respectively. Supernatants were collected at 12, 24, 48, 60 and 72 h post-infection, and viral titers were determined as log10 pfu/mL in MDCK cells. *P < 0.05, **P < 0.01, ***P < 0.005.

CEF and CBE cells were infected at the MOI of 0.01, which was 10-fold higher than that used to infect the MDCK cells (Figure 3). In MDCK cells, the r-06 strain had significantly higher virus yields at most time points. In CEF cells, r-06 strain reached the peak viral titer at 36 h, 12 h earlier than the r-06/NA2 strain did, and the r-06 strain had significantly higher virus yields at most of time points as well. In CBE cells, the r-06 strain reached the peak viral titer at 36 h whereas the r-06/NA2 strain did at 60 h. And the r-06 strain had significantly higher virus yields at three time points: 36, 48 and 60 h. Taken together, our data show that the r-06 strain had a higher replicative capacity than the r-06/NA2 strain in cell culture.

### Infectivity efficiency of r-06 and r-06/NA2 viruses in cell cultures

**Figure 4:**
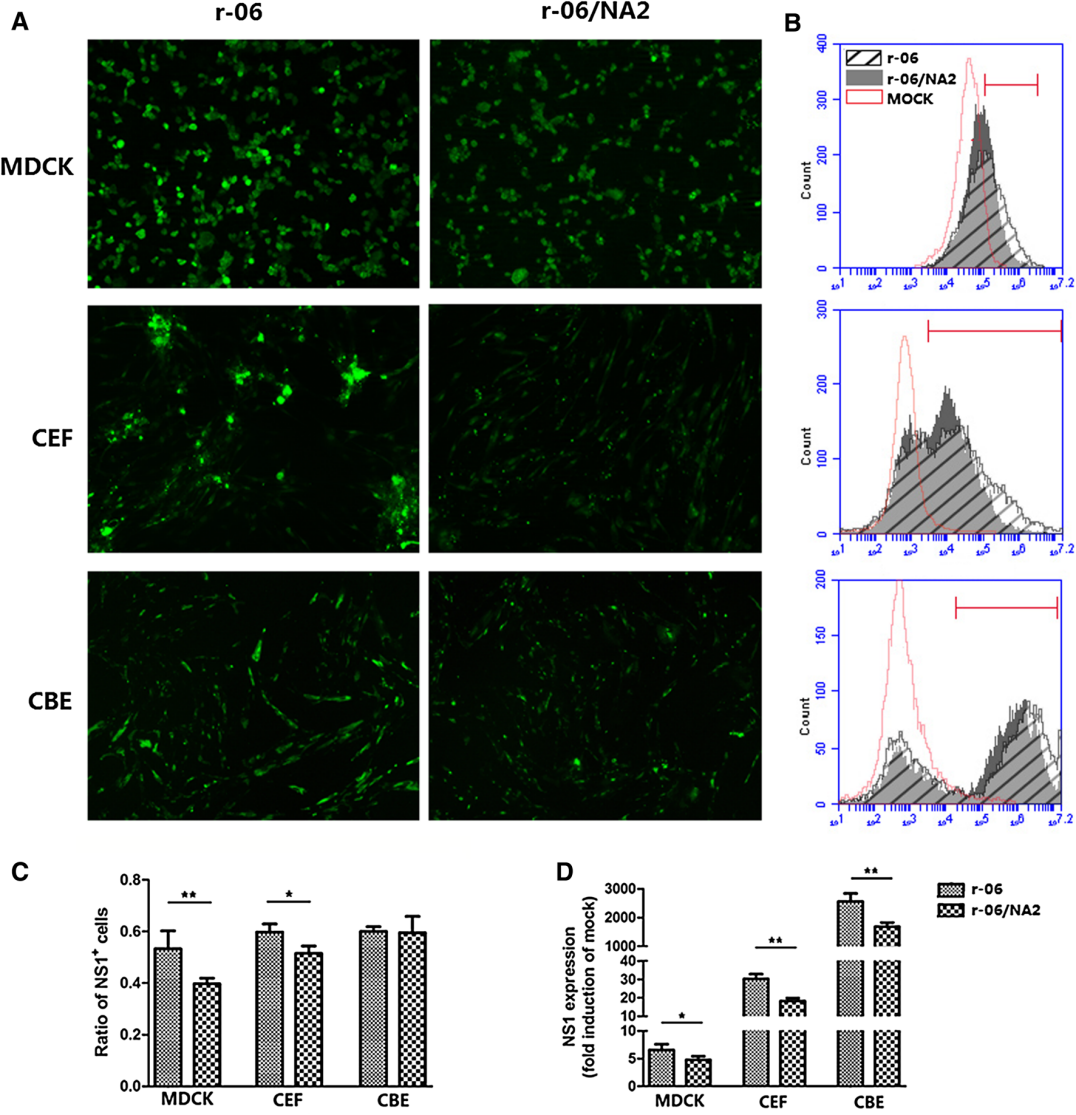
**Replication of r-06 and r-06/NA2 strains in MDCK, CEF and CBE cell cultures.** MDCK, CEF and CBE cells were infected with the two viruses at an MOI of 0.001, 0.01 and 0.01, respectively, and the viral NS1 expression was determined at 24 h after infection by immunofluorescence microscopy using an anti-NS1 antibody. **A** Indirect immunofluorescence staining of infected cells. **B** FACS analysis of infected cells. Red bar indicates the positive region. The infected cells in which fluorescence intensities (FIs) were larger than those in mock infected cells were confirmed as positive (right part of the mock histogram, red bar region). The area of red bar region represents the count of NS1^+^cells. **C** Quantification of the NS1^+^ proportion of infected cells in FACS analysis. NS1^+^ ratio was calculated by the proportion of cells of red bar region in all cells by CFlow plus software. **D** Quantification of NS1 expression by mean fluorescence intensities (MFIs) in FACS analysis. NS1 expression is depicted as fold MFI of mock-infected cells. MFIs of NS1^+^ cells (in red bar region) were calculated by CFlow plus software. *P < 0.05, **P < 0.01.

To evaluate the virus infectivity efficiency, viral NS1 expression was determined at 24 h after infection by immunofluorescence microscopy using an anti-NS1 antibody in all three cell lines. We inoculated confluent cell monolayers with r-06 or r-06/NA2 viruses, and positive cells exhibited green staining. From Figure 4A, we can see that in all three cells, stronger fluorescence was observed in r-06-infected cells than r-06/NA2-infected cells. Especially in MDCK cells, r-06 infection resulted in a noticeably greater number of stained cells than r-06/NA2 infection.

For quantification of the NS1-positive rate (%) and the mean fluorescence intensity (MFI) of NS1 expression in the NS1^+^ population, the three cell cultures infected with the two viral strains were subjected to flow cytometry (Figure 4B). NS1 expression was calculated by fold MFI of mock-infected cells. The proportion of r-06-infected MDCK cells (CIV NS1^+^) was 53.28 ± 3.46%, which was statistically significantly (*P* = 0.0097) higher than the proportion of r-06/NA2-infected cells (39.73 ± 1.08%) being consistent with the results obtained by immunofluorescence microscopy. Likewise, NS1 expression in r-06 infected cells was 6.327 ± 0.4923 times higher than that of mock-infected cells whereas r-06/NA2 infected cells was only 4.355 ± 0.0746 times higher (*P* = 0.0167). In CEF cells, the r-06 strain infected more cells than did the r-06/NA2 strain (59.77 ± 1.828% versus 51.50 ± 1.665%, *P* = 0.0288), as well as NS1 expression (28.94 ± 1.592 versus 17.89 ± 1.282 times, *P* = 0.0057). Interestingly, although the ratios of CBE cells infected by the two viral strains were similar, the NS1 expression levels were statistically significantly different (2658 ± 143.8 versus 1681 ± 81.15 times, *P* = 0.0041), being higher with r-06 infection (Figures 4C and D). Although the viral titer was the highest in MDCK cells, MFI of infected MDCK cells was fewer folds of mock infected cells than that of CEF or CBE cells. This phenomenon may be explained by the substantially much higher background staining for the MDCK cells. As we can see from Figure 4B, the MFI of mock-infected MDCK cells was 10^4.5^, while the MFI of mock-infected CEF or CBE cells was 10^3^.

### Enzymatic activity of NA of r-06 and r-06/NA2 viruses

To determine if the enzymatic properties of NA was affected by the 2-aa insertion in the NA stalk, we tested the NA activity against the single-valent substrate 4-MU-NANA and multivalent RBC substrate. The *K*m value that reflects affinity for the substrate showed no significant difference between r-06 and r-06/NA2 strains (7.884 ± 2.734 versus 4.815 ± 1.092 μΜ, *P* = 0.356). However, the *V*max value, which is determined by both the specific activity and the amount of enzyme in the reaction, was significantly higher in the r-06 strain as compared to the r-06/NA2 strain (28.31 ± 2.726 versus 14.81 ± 0.0926 FU/s, *P* = 0.009). The results indicate that the 2-aa insertion in the NA stalk could increase enzymatic activity.

### Virulence of r-06 and r-06/NA2 viruses in mice

**Figure 5:**
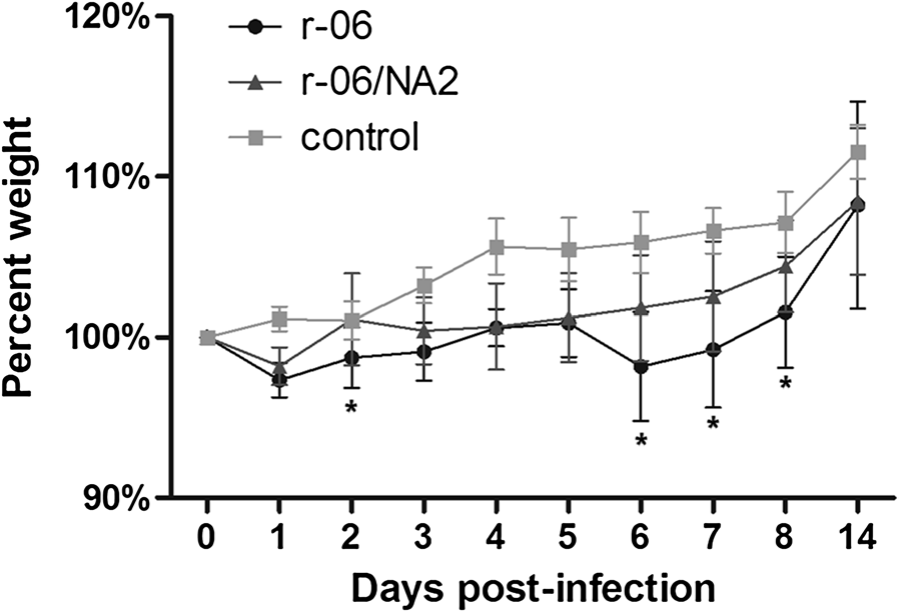
**Changes in body weight of BALB/c mice after inoculation with influenza virus strains r-06 (**  **) and r-06/NA2 (**  **) and PBS as control (**  **).** The percent of body weight change was calculated from body weights of 10 mice recorded daily after infection relative to that at day 0 (pre-infection). *P < 0.05.

In order to assess the differences in the pathogenicity of virus strains in mice, weight loss, viral loads in tissues and cytokine levels were compared. A total volume of 50 μL of the virus stock was inoculated per mouse with a viral titer of 2 × 10^6^ pfu/mL. Mice infected with the r-06 strain lost more body weight than those infected with r-06/NA2 during the challenge, especially at 2, 6, 7 and 8 dpi (*P* = 0.025, *P* = 0.014, *P* = 0.031, *P* = 0.038, respectively) (Figure 5).

A real-time PCR assay was performed to determine viral loads in the main tissues from CIV-infected mice. By 2 dpi, in both groups, virus had begun to shed in the feces and to replicate in the lung and intestine. By 4 dpi, the r-06 virus was detected in all tissues, whereas the r-06/NA2 virus could not be detected in the liver, kidney and brain. By 6 dpi, both infected groups had a high proportion of animals (5/5) with detectable viral RNA in all tested tissues. By 8 dpi, r-06 infected mice still exhibited a high level of detectable viral RNA in tissues, whereas r-06/NA2 infected mice showed a reduction. By 14 dpi, both groups presented signs of recovery, with some tissues having an undetectable virus level, however, r-06-infected mice showed fewer tissues cleared of virus. No viral RNA was detected in any tissues of mice from the control group (Table 1).

**Table 1:** **Proportion of mice in which viral RNA was detectable in samples**

Days post-infection (dpi)	Proportion of mice with detectable viral RNA (r-06, r-06/NA2)							
	Heart	Liver	Spleen	Lung	Kidney	Brain	Intestine	Feces
2	0/5,0/5	0/5,1/5	0/5,0/5	4/5,1/5	0/5,0/5	0/5,0/5	1/5,2/5	2/5,1/5
4	2/5,2/5	1/5,0/5	2/5,1/5	5/5,2/5	1/5,0/5	2/5,0/5	4/5,3/5	3/5,3/5
6	5/5,5/5	5/5,5/5	5/5,5/5	5/5,5/5	5/5,5/5	5/5,5/5	5/5,5/5	5/5,5/5
8	5/5,3/5	4/5,1/5	4/5,2/5	4/5,3/5	4/5,1/5	4/5,3/5	4/5,3/5	4/5,2/5
14	3/5,1/5	1/5,0/5	1/5,0/5	2/5,1/5	0/5,0/5	0/5,0/5	1/5,1/5	1/5,1/5

The data represent the results obtained from the mice inoculated with r-06 and r-06/NA2 strains, respectively.

**Figure 6:**
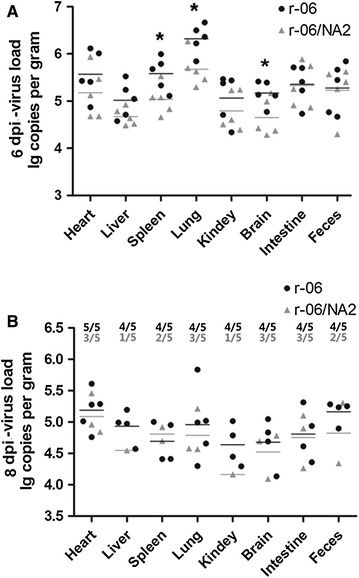
**Viral RNA loads in some tissues and feces of mice inoculated with r-06 (**  **) and r-06/NA2 (**  **) strains at 6 (A) and 8 dpi (B).** Five mice were chosen randomly and euthanized from each group. Every dot represents the viral RNA load in a tissue sample from one mouse. Viral loads are expressed as log10 RNA copy numbers per gram of sample. *P < 0.05.

Further, we compared viral RNA loads of tested tissues and fecal samples at 6 and 8 dpi (Figure 6). As shown in Figure 6A, at 6 dpi, viral RNA titers of spleen (*P* = 0.0357), lung (*P* = 0.0168), and brain (*P* = 0.0419) in the r-06-infected group were significantly higher than those in the r-06/NA2-infected group. At 8 dpi, viral RNA loads in all tissues from both infected groups were decreased as compared to those at 6 dpi, and r-06-infected mice appeared to have higher mean viral RNA loads in the detectable tissues than those of r-06/NA2-infected mice (Figure 6B). However, statistical analysis could not be performed because of an insufficient number (<3) of the detectable tissues in r-06/NA2-infected mice at 8 dpi.

**Figure 7:**
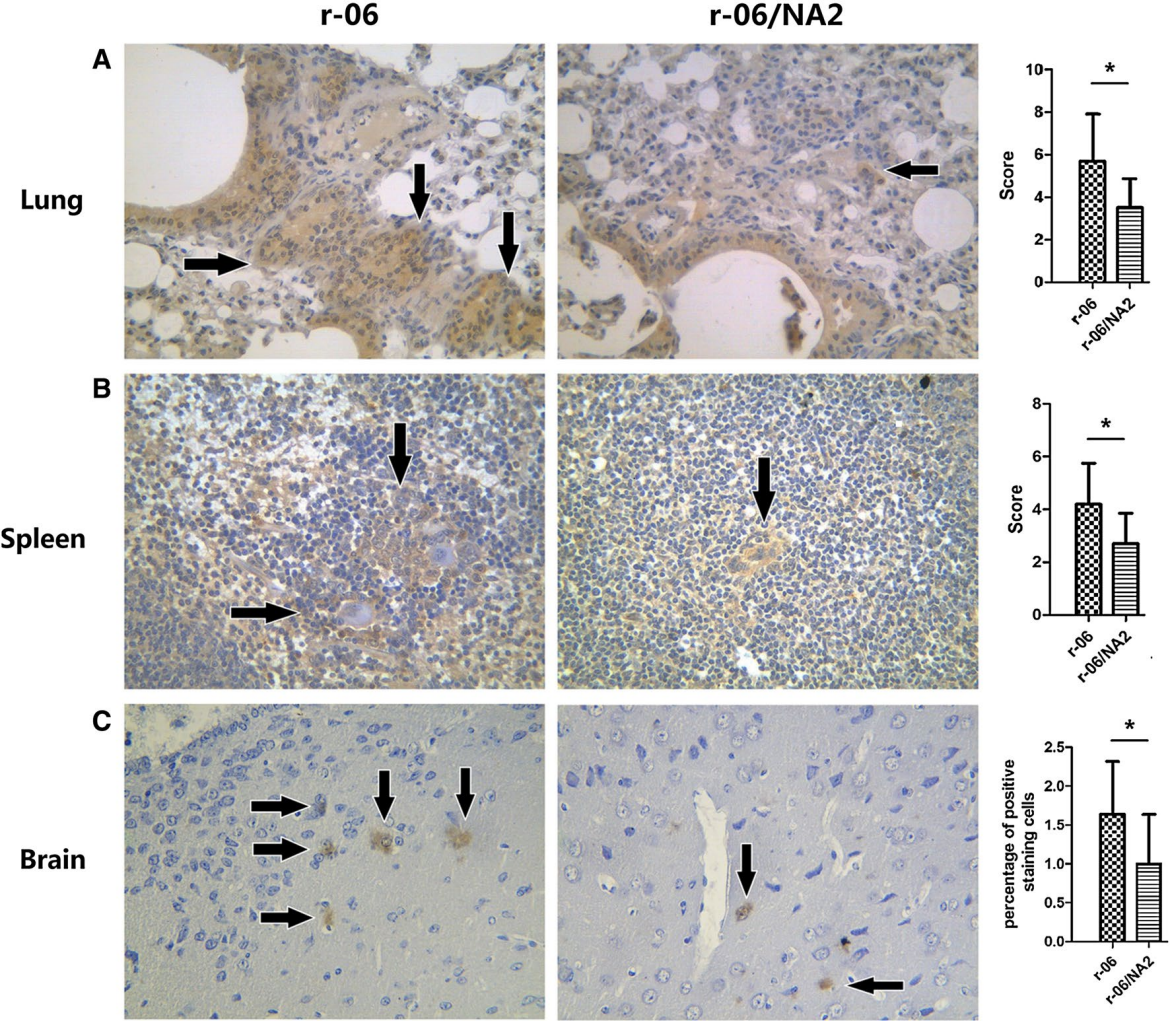
**Immunohistochemical detection of viral antigen and immunostaining assessment of lungs (A), spleens (B) and brains (C) of mice infected with r-06 and r-06/NA2 at 6 dpi.** Arrows indicate antigen staining. All the images are shown at 400× magnification. In the lung and spleen, scores (0–9) were assigned based on the percentage of positive staining and intensities of the signals. In the brain, the percentage of the positively stained cells was calculated *P < 0.05.

Considering that significantly higher viral RNA loads were detected in the lung, spleen and brain of r-06 infected mice, a quantitative assessment of immunostaining in the three tissues was performed for further verification of virus infection. In the lung and spleen, viral antigens could be detected in both infected groups, but compared to the r-06/NA2 group, significantly higher immunochemistry scores were observed in the r-06 group (*P* = 0.015 in lung and *P* = 0.025 in spleen, respectively). In the brain, antigen staining was much less than that in the lung and spleen from both infected groups, however, the percentage of positively stained cells in r-06-infected mice was still higher than that of r-06/NA2-infected mice (*P* = 0.045) (Figure 7).

**Figure 8:**
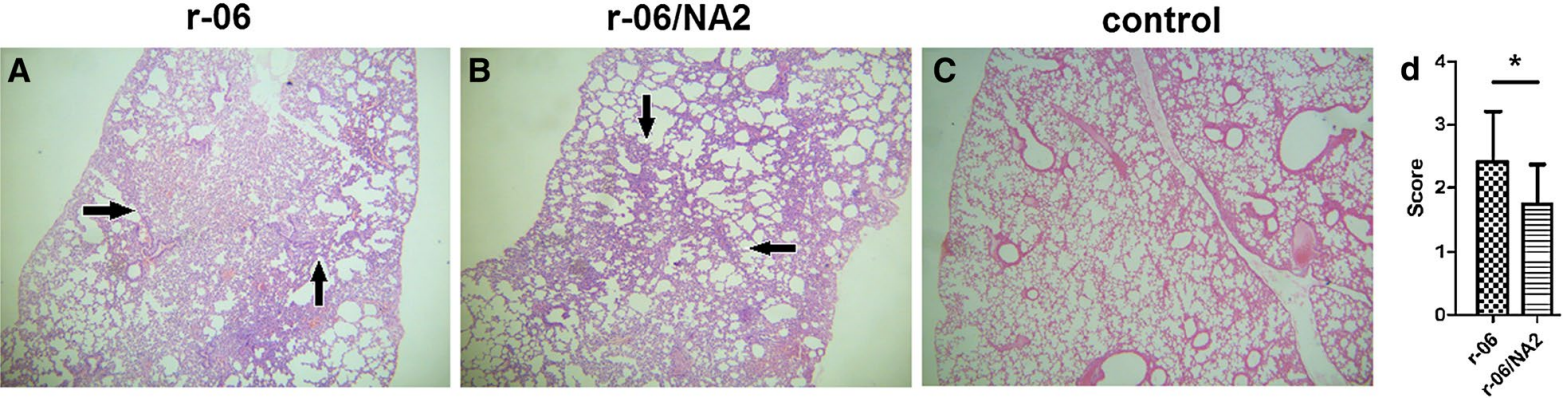
**Histopathological appearance of H&E-stained lung of mice infected with r-06 and r-06/NA2 at 6 dpi.** Interstitial pneumonia with alveolar septum thickened by the infiltration of a number of inflammatory cells (arrow) in r-06 (**A**) and r-06/NA2 infected mice (**B**). Normal morphology of lung in PBS-inoculated mice (**C**). All the images are shown at 40× magnification.

Histological lesions in the lungs were evaluated at 6 dpi. In r-06 or r-06/NA2-infected mice, interstitial pneumonia was obvious with the alveolar septum thickened by the infiltration of a number of inflammatory cells (Figure 8). However, r-06-infected mice showed larger areas of thickened alveolar septum. The pathological score of the lungs of r-06-infected mice was 2.417 ± 0.229, significantly higher (*P* = 0.0318) than that of r-06/NA2-infected mice (1.750 ± 0.179).

**Figure 9:**
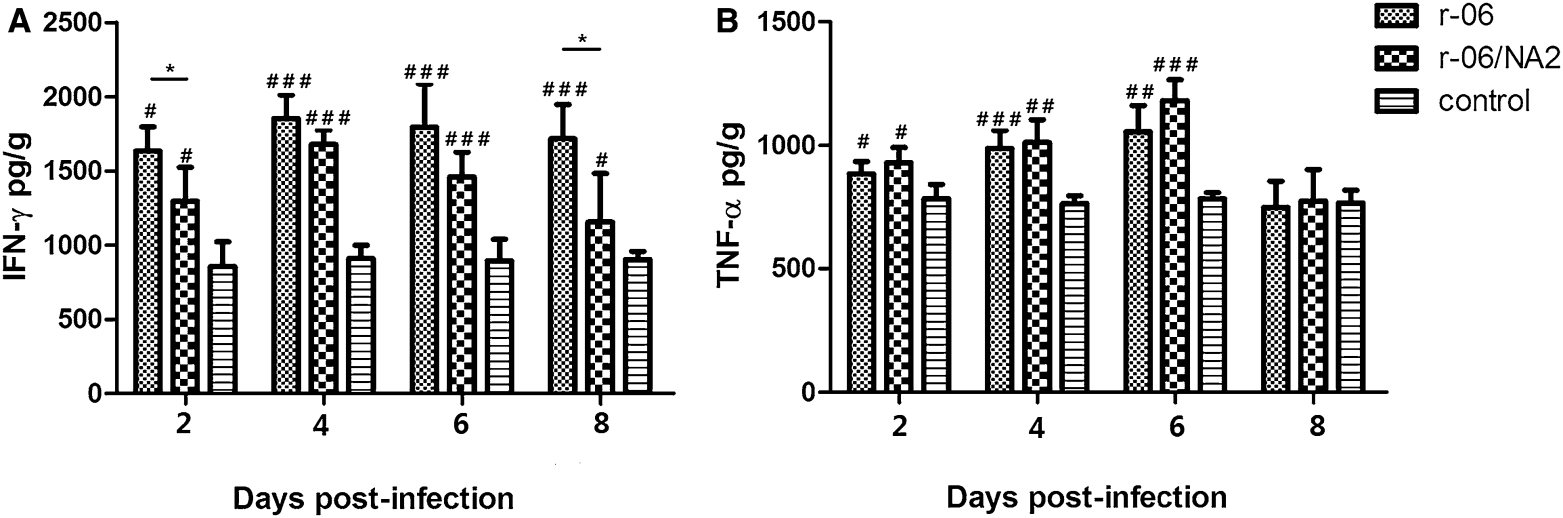
**Concentrations of IFN-γ (A) and TNF-α (B) in lungs of mice infected with r-06 (**  **) and r-06/NA2 (**  **) strains.** Concentrations of cytokines were determined via ELISA in lung supernatants taken from different days after infection. The results are expressed in terms of pg/g. *P < 0.05 indicates a significant difference for r-06 group compared with r-06/NA2 group. ^#^ *P* < 0.05, ^##^ *P* < 0.01, or ^###^ *P* < 0.005 indicates a significant difference between the infected groups and the PBS control group.

To further investigate if the two viral strains induced different immune responses, we determined the levels of two T helper type 1 (Th1) cytokines, IFN-γ and TNF-α, which both exert strong antiviral activity against influenza viruses. Both infected groups had statistically significantly higher levels of IFN-γ as compared to the control group at all time points, with a gradual increasing trend from 2 to 6 dpi. At 2 dpi, the r-06 infection promoted significantly higher levels of IFN-γ than r-06/NA2 did (*P* = 0.027), while both infected groups had the similar IFN-γ levels at 4 dpi and 6 dpi. At 8 dpi, the r-06 infected group still kept a higher level of IFN-γ while r-06/NA2 infected group had a decreasing trend, and IFN-γ levels in the r-06 group were significantly higher than those in the r-06/NA2 group (*P* = 0.023). For TNF-α, the two infected groups had statistically significantly higher levels than the control group at 2, 4 and 6 dpi, and then had reduced levels comparable to that of the control group at 8 dpi. No obvious difference was observed between the two infected groups in the level of TNF-α at any time point (Figure 9).

### Virulence of r-06 and r-06/NA2 viruses in chickens

To investigate whether the 2-aa insertion in the NA stalk would influence virus infectivity in SPF chickens, two groups of animals were inoculated intranasally with 100 μL (2 × 10^6^ pfu/mL) of r-06 or r-06/NA2 virus. As with the mouse infection experiment, we compared the results of body weight loss and viral RNA loads in various tissues and from oropharyngeal and cloacal swabs. Animals of both infected groups showed no clinical symptoms but grew more slowly than those of the control group, with reductions to the minimum weight at 3 dpi. The r-06/NA2-infected chickens grew more slowly over the first 6 days but faster from 7 dpi than r-06-infected chickens, but the differences between the two infected groups did not reach the statistical significance (*P* > 0.05) during the challenge period.

We found that in both infected groups the highest proportion of detectable viral RNA in organs and the widest range of tissue distribution were at 2 dpi. Additionally, the proportion of viral RNA positive organs in both groups decreased from 5 dpi, and the distribution range was reduced from 9 dpi. By 14 dpi, viral RNA could be detected in very few organs at a very low level. There were no apparent differences between the two viral strains in tissue distribution and proportion of tissues positive for viral RNA. A high rate (5/5) of viral RNA was detected at 2, 5 and 9 dpi in oropharyngeal swabs in both groups, and this frequency decreased (3/5) at 14 dpi. The virus-positive proportion in oropharyngeal swabs was higher than those in tissues (Table 2).

**Table 2:** **Proportion of chickens in which viral RNA was detectable in samples**

Days post-infection (dpi)	Proportion of chickens with detectable viral RNA (r-06, r-06/NA2)									
	Heart	Liver	Spleen	Lung	Kidney	Brain	Intestine	Pectoralis	Oropharyngeal swab	Cloacal swab
2	5/5;5/5	2/5;3/5	5/5;5/5	4/5;4/5	5/5;5/5	5/5;2/5	3/5;2/5	5/5;3/5	5/5,5/5	2/5,2/5
5	3/5;4/5	3/5;3/5	2/5;4/5	0/5;1/5	1/5;2/5	2/5;3/5	1/5;1/5	3/5;4/5	5/5,5/5	1/5,1/5
9	2/5;1/5	1/5;2/5	2/5;2/5	0/5;0/5	2/5;0/5	1/5;2/5	0/5;1/5	2/5;2/5	5/5,5/5	0/5,0/5
14	1/5;0/5	1/5;1/5	1/5;1/5	1/5;1/5	0/5;0/5	0/5;1/5	0/5;0/5	1/5;0/5	3/5,3/5;	0/5,0/5

The data represent the results obtained from the chickens inoculated with r-06 and r-06/NA2 strains, respectively.

**Figure 10:**
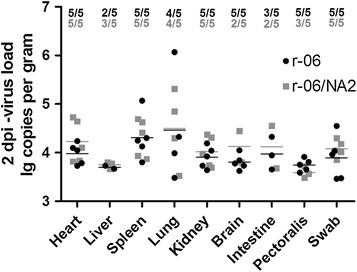
**Viral RNA loads in some tissues and swabs of chickens inoculated with r-06 (**  **) and r-06/NA2 (**  **) strains at 2 dpi.** Five chickens were chosen randomly and euthanized from each group. Each dot represents viral RNA load in an organ sample from one chicken. Viral loads are expressed as log10 RNA copy numbers per gram of sample.

We compared viral RNA loads in different tissues at 2 dpi since the widest range of tissue distribution and highest proportion of detectable viral RNA in tested tissues were found at that time point. Viral loads showed no significant differences between the two infected groups (Figure 10).

At 5 dpi, the lungs of the infected chickens were histologically characterized by interstitial pneumonia, similar to what had been observed in mice. However, the difference in the pathological scores of the lungs did not reach statistical significance between the two infected groups.

**Figure 11:**
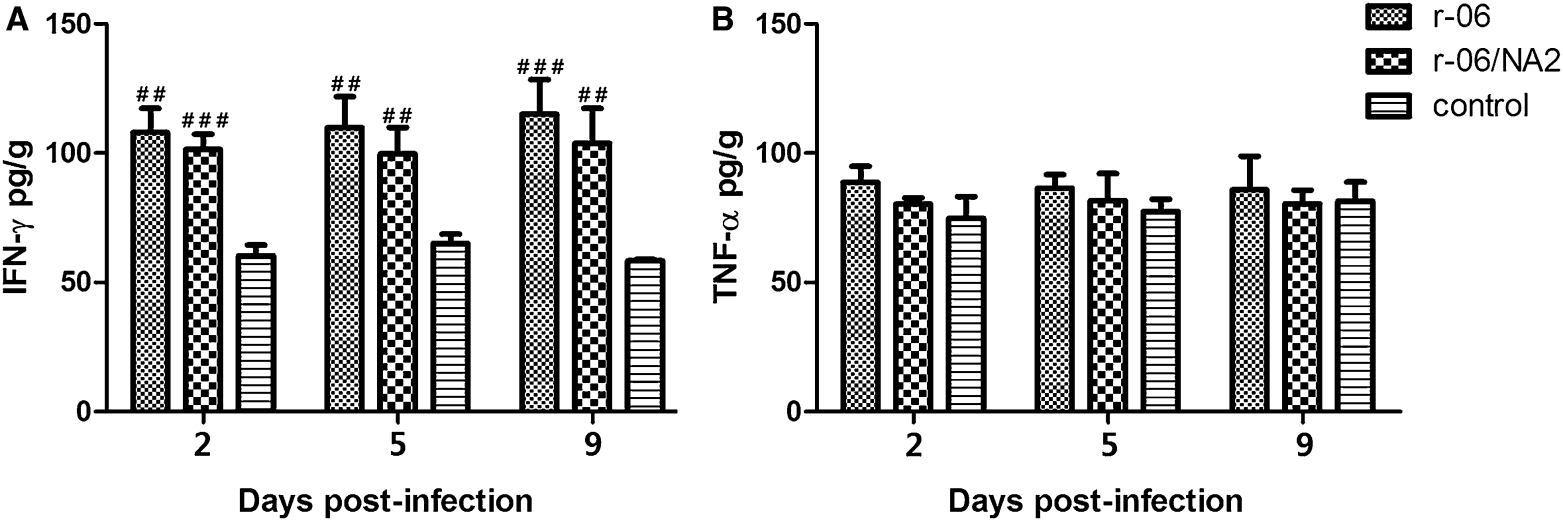
**Concentrations of IFN-γ (A) and TNF-α (B) in lungs of chickens infected with r-06 (**  **) and r-06/NA2 (**  **)**. Concentrations of cytokines were determined via ELISA in lung supernatants taken from different days after infection. The results are expressed in terms of pg/g. ^#^ *P* < 0.05, ^##^ *P* < 0.01, or ^###^ *P* < 0.005 indicates a significant difference between the infected groups and the PBS control group.

We also determined the levels of IFN-γ and TNF-α in chickens (Figure 11). Both infected groups showed statistically significantly higher levels (*P* < 0.05) of IFN-γ than the control group at 2, 5 and 9 dpi. For TNF-α, although both virus infection groups exhibited higher levels than the control group at 2 and 5 dpi, the difference did not reach statistical significance (*P* > 0.05). In comparing the two infected groups, the r-06 group showed higher levels of IFN-γ and TNF-α than the r-06/NA2 group at 2, 5, and 9 dpi, but no statistically significant difference was found (*P* > 0.05).

The sera of euthanized chickens were HI-tested for evidence of seroconversion on each scheduled day, and no difference was found between the two infected groups. Chickens of both groups showed no seroconversion (0/5) at 5 dpi, but all chickens had seroconverted (5/5) by 9 dpi with HI titers ranging from 2^2^–2^4^.

## Discussion

CIV is a newly identified, highly contagious respiratory pathogen of dogs. To date, H3N2 CIV have been reported across Asia. Our previous study found that several common mutations occurred at the receptor-binding sites, potential glycosylation sites and cleavage site in HA, and antigenic sites in both the HA and NA segments upon the initial interspecies transmission of H3N2 AIV to dogs [12]. In the following years, when H3N2 CIV were sequentially isolated in China, no important new common mutation occurred in HA, whereas a common 2-aa insertion in the NA stalk was found in all isolates in eastern and northern China since 2010. In particular, CIV in Guangdong showed an interesting pattern in that no insertion was detected from 2006 to 2011, and then one insertion and two insertions were found separately in 2012. Thus, NA of H3N2 CIV in Guangdong seemed to evolve from 2006 to 2012. NA is generally known to exhibit lower mutation rates than HA. However, during the prevalence of H3N2 CIV in China, the fact that a prominent sequence change was found in NA and not HA may indicate that this 2-aa insertion plays an important role in virus infectivity in dogs. However, as no genome sequences of H3N2 CIV from South Korea have been deposited in GenBank since 2013, we do not know whether the NA insertion has occurred yet in that country.

When AIV are transmitted from water fowl to poultry, they undergo genetic modifications that correlate with higher virulence and broader host range. Common genetic AIV mutations in viral proteins of poultry isolates are deletions in the stalk region of the NA and additions of glycosylation sites on HA [16]. These observations raise the question of whether the short NA stalk is more suitable for transmission of AIV to mammals. A recent study demonstrated that a further restriction to mammalian transmission of the majority of highly pathogenic avian influenza (HPAI) H5N1 viruses may be the short stalk length of the NA protein [23]. Therefore, we presume that the elongation of the NA stalk by 2 aa in nature may facilitate more efficient transmission of avian-origin H3N2 CIV in dog populations.

NA function, which is critical to virus release [7], may also act independently to determine the overall robustness of influenza virus and directly influence viral yield [24]. Previous studies reported that inserting at least more than 20 aa into the middle region of NA stalks can significantly increase the viral yield in both embryonated chicken eggs and MDCK cells [25, 26]. In our study, just a 2-aa insertion at the distal end of the NA stalk could lead to increased viral yield in MDCK, CEF and CBE cell cultures. In CEF and CBE cells, r-06 strain reached the peak viral titer earlier than the r-06/NA2 strain, which may be due to the fact that the 2-aa insertion facilitated the NA-mediated release of virus during budding from cells, thereby allowing the virus to infect neighboring cells more quickly and thus reach the peak titer earlier. In addition, in MDCK and CEF cells, after peak viral titers were reached, r-06/NA2 exhibited a continuous decreasing trend, whereas r-06 viral titer slightly increased from 60 to 72 h. This discrepancy may suggest that viable progeny viruses could still be produced in r-06 infected cells but fewer or not in r-06/NA2 infected cells at the late stage of infection. In MDCK, CEF and CBE cells, the mean fluorescence intensity of NS1 expression with r-06 infection was significantly higher than that with r-06/NA2 infection. This observation suggests that more progeny viruses were assembled in one single cell infected with long stalk virus. It is worth mentioning that in CBE cells, the two viral strains had similar infection rates at 24 h after inoculation, but the NS1 expression of r-06 was remarkably higher than that of r-06/NA2. This phenomenon presented here indicates that although the two viral strains infected the similar number of cells, the r-06 strain may replicate more efficiently in target cells.

The insertion increased virus infectivity and virulence not only in cell cultures but also in mice. Compared to r-06/NA2-infected mice, r-06-infected mice had higher viral RNA titers and more viral staining in the lung, spleen and brain at 6 dpi, higher frequencies of viral RNA-positive tissues at 8 dpi, and more severe lesions in the lung. The study from Matsuoka et al. [27] reported the contradictory observation that virulence in mice was enhanced for viruses with a truncated NA stalk compared to their equivalents with a long stalk. The conflicting results may be explained by the possibility that virulence is not due to the length of the NA stalk but rather to a new stalk motif constructed after the insertion or deletion of amino acids. Zhou et al. [28] concluded that a unique NA stalk motif is responsible for increased virulence and pathogenesis in mice, since neither an insertion nor deletion of aa in the NA stalk increased the virulence compared to the wild-type viral strain. Nevertheless, the insertion strain showed higher infectivity in MDCK cells and mice than the deletion strain, consistent with our findings. The decreased virulence of r-06/NA2 in mice may be attributed to the fact that a short-stalk NA is less competent in desialating the mucus barrier in the respiratory tract. Some previous studies [16, 23] demonstrated that a virus with a short NA stalk had a reduced ability to initiate infection in the presence of mucus, and neutralization of virus by mucus was significantly reduced through extending the NA stalk length. Influenza viruses require a careful balance of HA and NA activity in order to adapt to their hosts. Deletion of the NA stalk is often coupled with increased HA glycosylation as a compensation, which has been shown to reduce receptor binding affinity [29–32]. However, sequence analysis of H3N2 CIV strains with additional residues inserted in NA but no new common mutations in HA indicate that the increased infectivity by elongation of NA is independent of HA function. Our study shows that the CIV strain with elongated NA had increased virulence in mice with a more rapid and higher level of local IFN-γ response. An elevated level of IFN-γ observed in r-06-infected mice at 2 and 8 dpi may appropriately reflect the faster replication of the virus at this early period of infection. However, no obvious difference in TNF-α level was found between these two virus groups at this time. Sandbulte et al. [33] also found that differences in TNF-α induction by different viruses were less pronounced than those in interferon induction. A study from Seo and Webster [34] reported that TNF-α exhibited greater antiviral effects to counteract influenza virus infection than IFN-γ. Thus, although r-06/NA2 replicated more slowly, it could still trigger a high level of TNF-α as did r-06.

A shortened NA stalk has been reported to be a strong determinant of the adaptation and virulence of water fowl influenza viruses in chickens [16, 17]. Thus, the question arises as to whether the avian-origin H3N2 CIV with a 2-aa insertion in the NA stalk can adapt and increase virulence in chickens. However, our experimental infection in chickens shows the two viruses exhibited no obvious differences in body weight loss, viral loads of tissues and swabs, and levels of IFN-γ and TNF-α. We presume that the 2-aa insertion is not favorable for replication or transmission of H3N2 AIV in chickens. However, the insertion may lead to either a better transmission or higher virulence of H3N2 CIV in dogs, since the NA of AIV did not mutate and increase in length during many years in circulation until the interspecies transmission of H3N2 AIV to dogs and spread in China.

Notably, the viral load in chickens was lower than that in mice. This reduction in viral load may be caused by mutations in receptor binding sites, antigenic sites and/or glycosylation cleavage sites in HA [12]. These mutations may lead to H3N2 AIV that are not suitable for replication in chickens. In summary, this report showed that the 2-aa insertion at the distal end of the NA stalk region of H3N2 CIV could give rise to viruses that replicated more efficiently in MDCK, CEF and CBE cells and were more pathogenic in mice. These findings suggest that H3N2 CIV acquired an insertional mutation in NA and consequently achieved enhanced transmission to dogs. Multigenic adaptation facilitated H3N2 influenza viruses in overcoming the host species barrier and causing pandemics in dogs. As these viruses continue to evolve while spreading geographically, better surveillance and control strategies for the viral disease in companion animals should be developed.
